# ﻿Verification of two barracudina species *Lestrolepisjaponica* (Tanaka, 1908) and *L.philippina* (Fowler, 1934) (Aulopiformes, Paralepididae)

**DOI:** 10.3897/zookeys.1220.125860

**Published:** 2024-12-09

**Authors:** Hsuan-Ching Ho, Toshio Kawai

**Affiliations:** 1 Department and Graduate Institute of Aquaculture, National Kaohsiung University of Science and Technology, Kaohsiung, Taiwan National Kaohsiung University of Science and Technology Kaohsiung Taiwan; 2 Australian Museum, Sydney (Research Associate), Australia Australian Museum Sydney Australia; 3 Faculty of Fisheries Sciences, Hokkaido University, Hakodate, Japan Hokkaido University Hakodate Japan

**Keywords:** Biodiversity, ichthyology, ICZN, nomenclature, taxonomy

## Abstract

Examination of the type series of *Lestidiumjaponica* Tanaka reveals that the generally accepted concept of this species does not accord with the type series. A historical review of the literature showed that the characterization of *L.japonica* changed over time, and what has been recognized as “*L.japonica*” for more than 70 years actually represents a distinct and different species. Among the junior synonyms of “*L.japonica*”, *Paralepisphilippinus* Fowler, 1934 is resurrected as a valid species herein in a new combination, *Lestrolepisphilippina*. *Lestrolepisnigroventralis* Ho, Tsai & Li is recognized as a junior synonym of *L.japonica* herein. Revised diagnostic characteristics for both *L.japonica* and *L.philippina* are provided, along with comments on related names to verify their nomenclatural status.

## ﻿Introduction

Naked barracudinas, often classified as the Lestidiini, Lestidiinae, or sometimes Lestidiidae, are a group of small, slender fishes found worldwide from the surface to the deep sea. This group comprises seven genera with approximately 60 species, although some remain problematic (Ho et al. 2019a, 2019b; Ho pers. data). Among the genera, *Lestrolepis* can be distinguished from all others by having a small light organ (black dot) in front of the orbital margin and a ventral light organ divided into two branches, which are located inside the belly but visible through the translucent muscle. The genus *Lestrolepis* had few nominal species until recently when Ho and Golani (2019) and Ho et al. (2019a) reviewed and described several new species.

The taxonomic history of *Lestrolepis* is complex and somewhat confusing, even more so as the author, Robert Rees Harry (Harry 1953a, 1953b), later published under the name Robert R. Rofen (Rofen 1960, 1966). Three species have been commonly recognized in the previous literature, namely *Lestrolepisjaponica* (Tanaka, 1908), *Lestrolepisintermedia* (Poey, 1868), and *Lestrolepisluetkeni* (Ege, 1933). However, Ho and Golani (2019) suggested that *L.luetkeni* belongs to *Lestidiops* and resurrected *Lestrolepispofi* (Harry, 1953a), a name previously considered a junior synonym, as a valid name for the fish formerly referred to as *L.luetkeni*. Furthermore, Ho et al. (2019a) proposed that *L.intermedia* is restricted to the Atlantic Ocean and designated the western Pacific population as a new species, *Lestrolepisnigroventralis* Ho, Tsai & Li, 2019.

**Figure 1.**
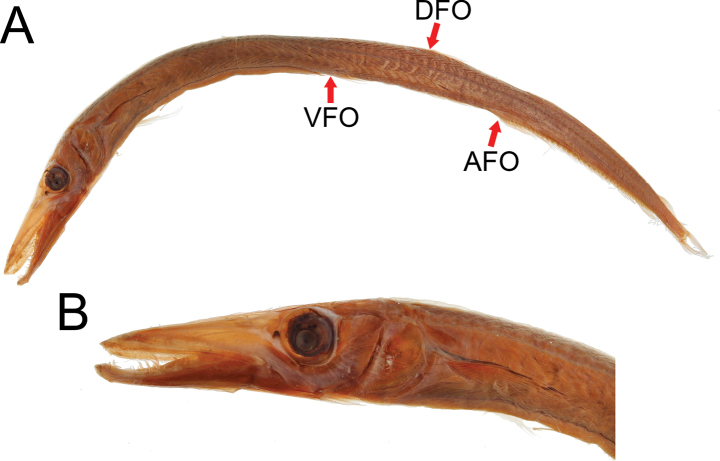
Holotype of *Lestidiunjaponicum* Tanaka, 1908 **A** lateral view; arrows point to origins of dorsal fin (DFO), pelvic fin (VFO), and anal fin (AFO) **B** lateral view of head.

**Figure 2.**
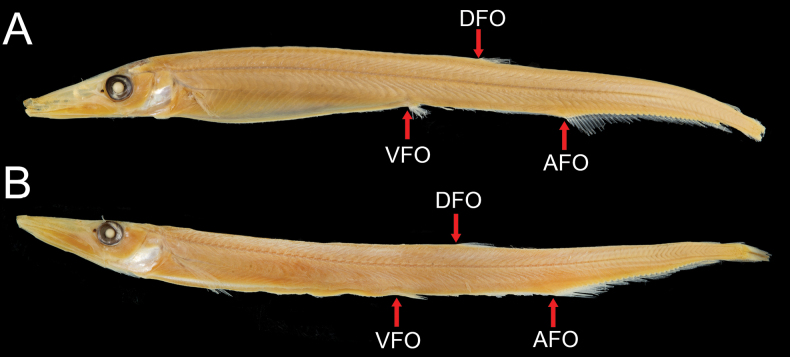
Types of *Paralepisphilippinus* (= *Lestrolepisphilippina*) **A** holotype, USNM 92323, ca 118 mm SL**B** paratype, USNM 93414, 126 mm SL; arrows point to origins of dorsal fin (DFO), pelvic fin (VFO), and anal fin (AFO).

Recently, the second author (TK) examined specimens deposited in the fish collection of Hokkaido University and identified inconsistencies between the original description and the specimens currently recognized as *L.japonica*. Examination of the holotype (Fig. 1) and paratype of *L.japonica* showed them to be indistinguishable from *L.nigroventralis*, suggesting that the latter should be regarded as a synonym of *L.japonica* and that the species previously generally referred to as “*L.japonica*” requires a new name. The first author (HH) examined the type series of *Paralepisphilippinus* (Fig. 2), previously regarded as a junior synonym of *L.japonica*, and found them to be identical to what was commonly recognized as *L.japonica* in the western Pacific Ocean. Additionally, several other names were implicated in the synonymy or misidentifications under these names (Table 1).

**Table 1.** Verification of some name records of *Lestrolepis* and their current status.

Publication	Name used	Correct names
Poey 1868	*Paralepisintermedius*	Valid as *Lestrolepisintermedia*
Tanaka 1908	*Lestidiumjaponicum*	Valid as *Lestrolepisjaponica*
Ege 1933	*Paralepisluetkeni*	Valid as *Lestidiopsluetkeni*
Ege 1933	*Paralepisbellottii*	Synonym of *Lestidiopsluetkeni*
Fowler 1934	*Paralepisphilippinus*	Valid as *L.philippina*
Fowler 1944	*Sudisvanderbilti*	Synonym of *L.intermedia*
Harry 1953a	*Lestidiumjaponicum*	*L.philippina*
Ege 1953	*Lestidiumintermedium* (in part)	*L.japonica*
Kamohara 1955	*Lestidiumjaponicum*	*L.japonica*
Rofen 1966	*Lestrolepisjaponica*	*L.philippina*
Fujii 1984	*Lestrolepisjaponica*	*L.philippina*
Fujii 1984	*Lestrolepisintermedia*	*L.japonica*
Nakabo 2000, 2002	*Lestrolepisjaponica*	*L.philippina*
Kim et al. 2007, 2020	*Lestrolepisjaponica*	*L.philippina*
Ho et al. 2019a	*Lestrolepisnigroventralis*	Synonym of *L.japonica*
Ho et al. 2019a	*Lestrolepisjaponica*	*L.philippina*

To clarify the identification of these species, we conducted a study on the type series of *Lestrolepisjaponica* and *L.philippinna* and provide here revised diagnoses or comments for the species related to these names. In the present study we confirm the validity of *L.japonica*, synonymize *L.nigroventralis* with *L.japonica*, resurrect *L.philippina*, and clarify the taxonomic history of these species.

## ﻿Materials and methods

Methods for taking morphometrics and meristics followed Ho et al. (2019a). Most morphometric and meristic data are adopted from Ho et al. (2019a), with those of additional specimens examined.

### ﻿Abbreviations

**SL**, standard length; **HL**, head length; **TL**, total length; **VFO**, **DFO**, **AFL**, the origins of pelvic fin, dorsal fin and anal fin, respectively; **V–D**, space between VFO and DFO; **V–A**, space between VFO and AFO. Other abbreviations followed Ho and Lin (2023).

## ﻿Results

### ﻿Genus *Lestrolepis* Harry, 1953

On the taxonomic issues

*Lestidiumjaponicum* was described based on two types collected from Sagami Bay (ZMUT 2013 and 2014). In the synonymy of this species, Harry (1953a) included *Paralepisbellottii* Ege, 1933 as a queried synonym and *Paralepisphilippinus* Fowler, 1934 as a junior synonym of *L.japonicum*. Subsequently, Harry (1953b) re-assigned *japonicum* to *Lestrolepis*, and this placement in *Lestrolepis* has been widely accepted by subsequent authors.

Post (1972) suggested that *L.japonica* “possibly is a synonym of *Lestrolepisintermedia* Poey, 1868". Examination of the holotype of *L.japonica* revealed that it is indeed similar to *L.intermedia* but that it differs in several characters. Consequently, specimens recognized as “*Lestrolepisjaponica*” in much of the literature belong to another species that requires a name, and the following two names listed as synonyms of “*japonicum*” by Harry (1953a) should be considered as candidates for this species.

The original description of *Paralepisbellottii* was based on a single specimen (60 mm SL, 64 mm TL). This species was synonymized with *Lestidiumnudum* Gilbert, 1905 and *Paraelpisluetkeni* Ege, 1933 by Ege (1953:52). Rofen (1960: 206) synonymized the name with “*Lestidiumleutkeni*” and Rofen (1966) later placed it in the genus *Lestrolepis*. The first author examined the holotype of *bellottii* and found it to be very similar to the holotype of *luetkeni*. Type specimens of both are also different from *Lestidiumnudum*, rejecting the synonymy proposed by Ege (1953). In fact, *luetkeni* is placed in the genus *Lestidiops* by Ho and Golani (2019), and the status of *bellottii* will be discussed in another work prepared by HH.

The original description of *Paralepisphilippinus* was based on eight type specimens collected from the Philippines. Examination of the type series revealed that it is different from that of *L.japonica* and is what has been commonly recognized as “*L.japonica*” in the western Pacific Ocean.

Consequently, neither *philippinus* nor *leutkeni* or *bellottii*, can be regarded as synonyms of *japonica*, but can be considered here to represent what has been called “*L.japonica*” in the western Pacific (Table 1). The oldest name, *philippina* is herein resurrected as valid in a new combination *Lestrolepisphilippina* (Fowler, 1934) for this species.

Ho et al. (2019a) described *Lestrolepisnigroventralis* to accommodate the population of what has been called “*Lestrolepisintermedia*” in the western Pacific Ocean, i.e. Japan, Korea, and Taiwan, etc. As the type series is the same as that of *L.japonica* (Table 1), this name is now recognized as a junior synonym of *L.japonica*. In addition, the specimens recognized as *L.japonica* by Ho et al. (2019a) are now re-identified as *L.philippina*.

#### ﻿Records of *Lestrolepisjaponica* and *L.intermedia* in the western Pacific

As mentioned above, Harry (1953a) mistakenly considered *Lestrolepisphilippina* as a junior synonym of *L.japonica* without examining the vertebral counts of the type series of *L.japonica*. This oversight led to a change in the definition of *L.japonica* based on the type series of *L.philippina*, resulting in subsequent effects. For instance, Rofen (1966: 381) differentiated *L.intermedia* from *L.japonica* based on different vertebral numbers (91–98, vs 84–89), indicating that his *L.japonica* was actually *L.philippina*. Subsequent literature records of *L.japonica* following Harry’s definition recognized those with fewer vertebrae as *L.japonica* (see synonymy below).

The earliest records of *Lestrolepisintermedia* in the Western Pacific Ocean are found in Ege (1953), who documented juveniles of *L.intermedia* collected from various locations worldwide, including Japan, Taiwan, and the Philippines (also see Rofen 1966). However, subsequent publications in the western Pacific Ocean (e.g., Matsubara 1955) did not evidently document this name.

Fujii in Masuda et al. (1984:77) provided a brief description of *Lestrolepisintermedia* and mentioned that “*L.intermedia* and *L.japonica* are often collected by shrimp-trawl net in Suruga Bay”. Fujii also provided total vertebrae counts of 95–97 and total lateral-line scales of 74–81 for his *L.intermedia*, and 84–87 and 62–69, respectively, for his *L.japonica*. These definitions for both species evidently were followed by all subsequent publications in Japan and nearby areas (e.g., Nakabo 1993, 2000, 2002; Shen et al. 1993, Kim et al. 2007), while Ho et al. (2019a) went further and mistakenly recognized the western Pacific population of *L.intermedia* as a new species, *L.nigroventralis*.

##### ﻿ Lestrolepis japonica

Taxon classificationAnimaliaAulopiformesParalepididae

(Tanaka, 1908)

F167DE90-1FBC-58EC-8A41-506F9CE3A065

**Figure 3.**
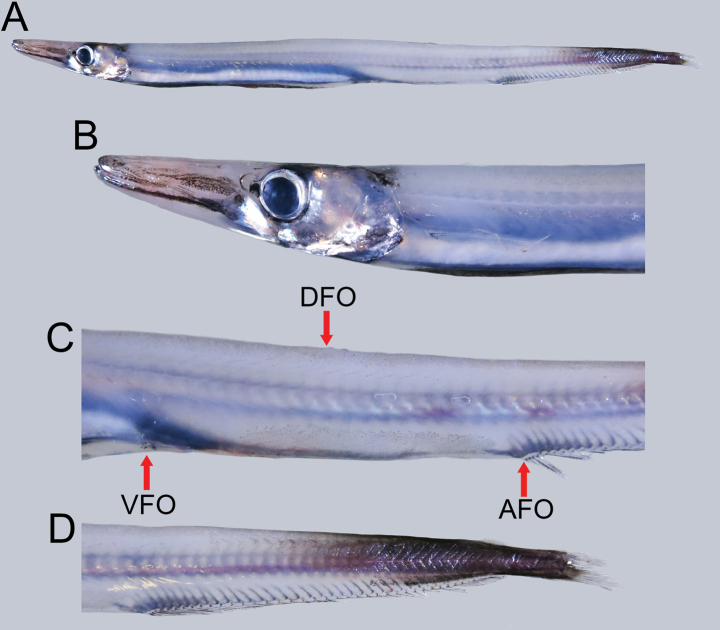
Fresh condition of *Lestrolepisjaponica* (Tanaka, 1908), NMMB-P027930, 244 mm SL**A** lateral view **B** lateral view of head **C** lateral view of body showing origin of dorsal fin (DFO), pelvic fin (VFO), and anal fin (AFO) **D** lateral view of caudal region.

Figs 1 , 3 , Table 2

Lestidium japonicum Tanaka, 1908:27 (type locality: Sagami Sea, Japan); Jordan et al. 1913: 50 (Sagami Sea, Japan; list); Okada and Matsubara 1938: 61 (Sagami Sea, Japan; key); Matsubara 1941: 8 (in part: Japan); Matsubara 1955: 262 (in part: Sagami Sea, Wakayama and Mie, Suruga Bay, Japan; key); Matsubara 1963: 262 (in part: Sagami Sea, Wakayama and Mie, Suruga Bay, Japan; key).
Lestrolepis intermedia (non Poey, 1868): Fujii in Masuda et al. 1984:77 (Japan; short description); Chen and Yu 1986:324 (Taiwan; synopsis); Nakabo 1993:319 (Japan, picture key); Shinohara et al. 1996:164 (Honshu; list); Nakabo 2000:371 (Japan, picture key); Nakabo 2002:371 (Japan, picture key); Shinohara et al. 2005:409 (Ryukus Is.; list); Kim et al. 2007:63 (Korea; new record); Ikeda and Nakabo 2015: 312 (Japan; short description).
Lestrolepis nigroventralis Ho, Tsai & Li, 2019:123 (type locality: off Dong-gang, Pingtung, southwestern Taiwan).

###### Status of the holotype.

Tanaka (1908) indicated that the holotype of his *Lestidiumjaponicum* was registered as 2013 (now ZMUT 2013) with a length of 174 mm SL, and the paratype (in the Table, without a catalog number) was 190 mm SL. Regardless, Harry (1953a) gave the holotype as ZMUT 2014, and the paratype as ZMUT 2013. Notwithstanding, Post (1972) documented the types and recognized ZMUT 2013 as the holotype and ZMUT 2014 as the paratype. We examined both types and re-measured the holotype (ZMUT 2013) as 159.5 mm SL and the paratype (ZMUT 2014) as approximately 180 mm SL, the latter being in poor condition.

###### Synonym name.

*Lestrolepisnigroventralis* was described to accommodate the Pacific population of *L.intermedia*. However, because of its nearly identical morphology (Table 2), it is now recognized here as a junior synonym of *L.japonica*. The following data are mainly derived from types and non-types of *L.nigroventralis*, combined with the type series examined by us.

**Table 2.** Selected morphological and meristic data of *L.japonica* and *L.philippina*. * Data of “*L.nigroventralis*” and ** of “*L.japonica*” taken from Ho et al. (2019a). HT = holotype; PT = paratype(s).

	*L.japonica*				*L.philippina*		
	Types		Types of *L.nigroventralis*		Types		Non-types**
	HT	PT	HT	Types*	HT	PT	
SL (mm)	159.5	ca 180	230	180–249 (*n* = 11)	118.2	109.6–128.0 (*n* = 4)	88.0–222 (*n* = 29)
Proportion (%)				Mean (Range)			Mean (Range)
HL/SL	20.9	21.1	20.0	19.6 (18.9–20.7)	20.8	21.3–22.0	20.5 (18.0–21.8)
BD/SL	5.7	5.2	5.3	5.2 (4.9–5.6)	8.5	7.1–7.9	6.9 (5.9–8.6)
PreD/SL	63.7	–	62.8	62.7 (61.7–63.3)	58.8	59.4–60.9	61.8 (60.2–63.5)
PreV/SL	52.7	–	52.1	51.6 (50.6–52.6)	51.2	51.6–53.4	53.0 (52.3–55.2)
PreA/SL	73.3	–	73.5	73.3 (71.9–74.8)	72.8	73.0–75.2	74.9 (75.8–76.6)
ED/HL	20.1	17.9	15.5	16.1 (14.8–17.3)	19.1	18.1–19.1	18.4 (15.9–22.6)
SN/HL	54.7	53.8	52.4	54.4 (52.4–57.0)	52.8	49.8–52.8	50.8 (47.7–53.4)
HD/HL	27.1	24.8	26.3	26.4 (25.4–27.4)	30.9	27.8–30.8	28.5 (28.6–28.5)
UJ/HL	51.4	49.6	49.1	49.0 (47.2–50.4)	47.6	47.7–49.5	46.8 (44.0–50.0)
V–D/V–A	53.1	51.0	50.0	51.0 (47.6–55.0)	35.3	34.0–36.1	39.1 (32.5–43.1)
**Meristics**							
Anal-fin rays	42	ca 40	42	41–43	ca 38	37–38	36–40
Vertebrae							
PHV	34	33	33	32–35	29	30–31	29–32
PVV	33	34	34	33–35	30	30–32	30–33
PDV	43	44	44	43–46	38	38–39	36–40
PAV	53	55	55	53–57	48	49–51	48–51
CV	60	61	62	60–66	55	56–59	52–58
TV	94	95	95	94–98	85	86–89	84–88
V–D	9	10	10	9–11	8	7–8	5–9
Lateral-line scales							
PVLL	34	–	34	33–36	30	30–32	31–32
PDLL	44	–	44	43–46	38	39–40	38–40
PALL	54	–	54	53–57	49	49–51	49–50
TLL	ca 81	–	78	75–81	71	68–70	65–72

###### Distinguishing features.

A species of *Lestrolepis* with DFO situated at about midline of V–A, V–D 46.8–55.0% of V–A; anal-fin rays 41–43 (mainly 41–42); lateral-line scales: PVLL 33–36 (34–35), PDLL 43–46 (44–46), PALL 53–57 (55–56), TLL 75–81 (77–80); vertebral counts: PHV 32–35 (mainly 32–34), PVV 33–35 (34–35), PDV 43–46 (44–45), PAV 53–57 (54–55), CV 60–66 (61–64), TV 94–98 (94–97); vertebrae between DFO and VFO 9–11. Body slender, body depth at pectoral-fin base 15–19 times in SL; band of melanophores along abdominal margin. Attains 249 mm SL.

###### Distribution.

Known from the northwestern Pacific Ocean off Japan, Taiwan, and Korea. Records from other localities require verification.

###### Remarks.

The taxonomic concept of what has long been called *Lestrolepisjaponica* is now revised based on the type series and additional specimens. A review of the literature with documentation of *L.japonica* is listed in the synonymy above, although there might be more records that are not included therein. Detailed comparisons with congeners are provided in Ho et al. (2019a, as *L.nigroventralis*).

There are some minor differences found in the types compared to the non-types (Table 2). The head length is slightly larger (20.9–21.1%, vs 18.9–20.7% SL); the predorsal length is slightly larger in the holotype (63.7%, vs 61.7–63.3% SL; not available for the paratype); the eye diameter is slightly larger (17.9–20.1%, vs 14.8–17.3% HL); the upper jaw of the holotype is slightly longer (51.4%, vs 47.2–50.4% HL). These proportional measurements may be attributed either to population variation or due to long-term preservation of the types. The meristic values of types fall entirely within the range of non-types (Table 2).

##### ﻿ Lestrolepis philippina

Taxon classificationAnimaliaAulopiformesParalepididae

Fowler, 1934

C7778AD4-FCF0-57A7-81E9-F050C47F842C

**Figure 4.**
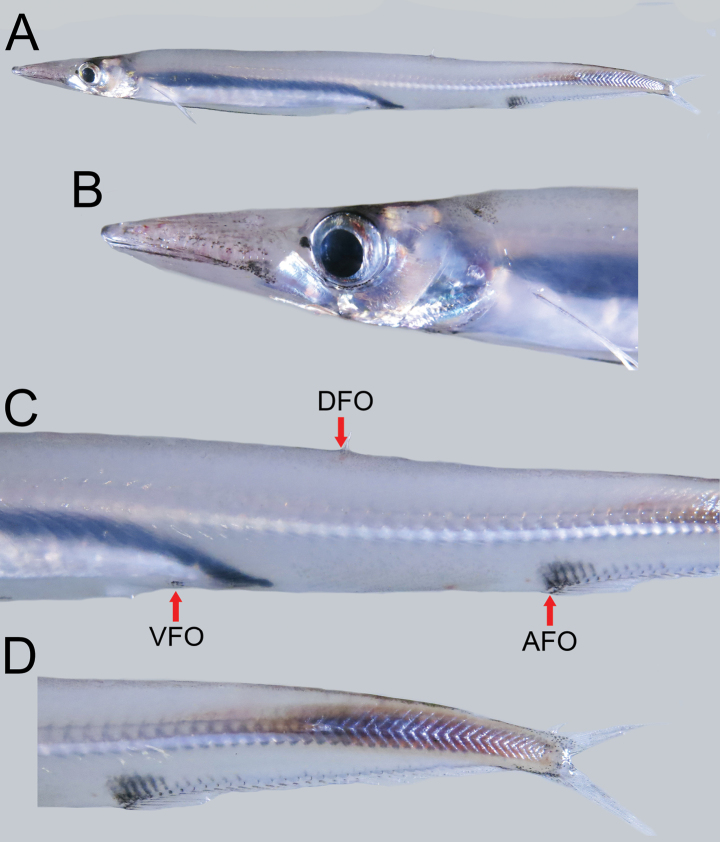
Fresh condition of *Lestrolepisphilippina* (Fowler, 1934), NMMB-P027934 (1 of 17), ca 175 mm SL**A** lateral view **B** lateral view of head **C** lateral view of body showing origin of dorsal fin (DFO), pelvic fin (VFO), and anal fin (AFO) **D** lateral view of caudal region.

Figs 2 , 4 , Table 2

Paralepis philippinus Fowler, 1934: 281, fig. 42 (type locality: Varadero Harbor, Philippines).
Lestidium japonicum (not of Tanaka): Matsubara 1941: 8 (in part: Japan); Matsubara 1955: 262 (in part: Sagami Sea, Wakayama and Mie, Suruga Bay, Japan; key); Matsubara 1963: 262 (in part: Sagami Sea, Wakayama and Mie, Suruga Bay, Japan; key).
Lestrolepis japonica (not of Tanaka): Fujii in Masuda et al. 1984:77 (Japan; short description); Nakabo 2000:371 (Japan; picture key); Paxton in Randall and Lim 2000:592; Nakabo 2002:371 (Japan; key); Shinohara et al. 2005:409 (Honshu; list); Kim et al. 2007:64 (Korea; new record); Ikeda and Nakabo 2015: 312 (Japan; short description); Motomura et al. 2017:51; Ho and Golani 2019:578 (mentioned); Ho et al. 2019a:127 (Taiwan and Japan; description); Kim et al. 2020:67; Misawa et al. 2020:273.

###### Taxonomy.

Fowler (1934) originally described *Lestrolepisphilippina* from the Philippines. Subsequently, Harry (1953a) synonymized *L.philippina* with *L.japonica*, providing a description based on the type series of *L.japonica*, *L.philippina*, and additional specimens. Although the name *L.philippina* was not mentioned in subsequent literature, it is likely that many records recognized as *L.japonica* are, in fact, misidentifications of *L.philippina*. In a more recent study, Ho et al. (2019a) presented a detailed description of what was previously identified as *L.japonica*, now re-identified as *L.philippina*.

###### Distinguished features.

A species of *Lestrolepis* with DFO situated well before midline of V–A, V–D 32.5–43.1% of V–A; anal-fin rays 36–40 (mainly 37–39); lateral-line scales: PVLL 30–32 (31), PDLL 38–40 (38–39), PALL 49–51, TLL 65–72 (65–68); vertebral counts: PHV 29–32, PVV 30–33 (30–32), PDV 36–40 (37–40), PAV 48–51, CV 52–58 (54–57), TV 82–88 (84–88); vertebrae between DFO and VFO 5–9 (7–8). Body moderately slender, body depth at pectoral-fin base 13–16 times in SL; narrow band of melanophores along abdominal margin. Attains 222 mm SL.

###### Distribution.

Widespread in the western Pacific Ocean, with confirmed records from Japan, Taiwan, the Philippines, northwestern Australia, and the South China Sea.

###### Remarks.

Some minor differences are observed between the holotype and non-types (Table 2). The predorsal length (58.8%, vs 60.2–63.5% SL), preventral length (51.2%, vs 52.3–55.2% SL), and preanal length (72.8%, vs 75.8–76.6% SL) are slightly smaller in the holotype compared to paratypes and non-types. The head depth is slightly larger (30.9% SL) compared to that of paratypes and non-types (27.8–30.8% SL). These differences may be attributed to individual variation or long-term preservation effects. The meristic values of the type series fall well within those of the non-types (Table 2).

## ﻿Discussion

Several ambiguities in earlier studies have led to subsequent problems, including the inadvertent naming of a new species. Tanaka (1908) described *Lestidiumjaponicum* with 42 anal-fin rays in the holotype and 49 in the paratype. Harry (1953a) counted 42 and 41, respectively, for the same individual (note that Harry switched the holotype and paratype). Jordan et al. (1913) and Okada and Matsubara (1938) included *Lestidiumjaponicum* in their species list as known from Sagami Sea. On the other hand, Hubbs (1916) cited Tanaka (1908) and gave 42–49 anal rays. Parr (1928) noted that *Lestidiumjapponicum* [sic] is only known from the coast of Japan.

Matsubara (1941) reported two specimens (178.8 mm SL and 102.2 mm SL) of this species collected from Suruga Bay, Japan, the former specimen having 43 anal-fin rays, and the latter 35. Harry (1953a: 187) suggested the first one is *Lestidiumjaponicum*, whereas the latter was unknown. We concur that the former is *Lestrolepisjaponica*, and the latter is *L.philippina* based on their counts of anal-fin rays (cf. 40–43 in *L.japonicus* and 36–40 in *L.philippina*; Table 2). Matsubara (1941) also mentioned that *japonicum* closely resembles *L.philippinus*. This could have been the beginning of confusion of the species (see also Matsubara 1955, 1963).

Harry (1953a) reported examining the types and additional specimens of *L.japonica* and identified *L.philippina* as a junior synonym. However, given clear differences, such as the position of DFO, the relatively slender body, snout, and jaws, as well as different vertebral and lateral-line counts, it remains uncertain why Harry considered them senior synonyms of *L.philippina*, perhaps being influenced by Matsubara (1941). It seems likely that Harry never examined radiographs of the types of *L.japonica*; otherwise, he might have noticed the distinctly high vertebral counts in these two specimens.

Ege (1953) documented juveniles of *Lestrolepisintermedia* (as *Lestidiumintermedium*) from the western Pacific, i.e. Japan, Taiwan, and the Philippines, etc. Assuming he identified these specimens correctly, for example, regarding the position of DFO, his specimens should be *L.japonica* in this work. However, he never considered the names “*japonicum*” or “*philippinus*” in his publication. Based on the museum collection (Ho pers. obs.), *L.philippina* is far more abundant compared to *L.japonica*, and it remains unknown why Ege did not recognize such a distinct form. It is notable that Ege (1953) recognized from this region *Lestidiumnudum*, which has similar fin position. It is likely Ege misidentified *L.philippina* as *Lestidiumnudum*, because the latter is more or less restricted to the Hawaiian Islands and the central Pacific (Ho pers. data).

Fujii in Masuda et al. (1984) appears to have followed Harry’s (1953b) definition and recognized the population with few vertebrae as *L.japonica*, which is now confirmed to be a misidentification of *L.philippina*. He also identified *L.intermedia* from Japan and provided a short description. His provided counts of 95–97 total vertebrae and 74–81 lateral-line scales indicate that his description was, in fact, that of *L.japonica*.

Regarding other paralepidid species, some taxonomic problems have been reviewed, such as the resurrection of *Lestrolepispofi* and the placement of *L.leutkeni* in *Lestidiops* (Ho and Golani 2019). Matching adults to these species with only juveniles known, or redescriptions of species have been undertaken (Ho and Huang 2022a, 2022b; Ho and Lin 2023; Ho and Tsai 2023). However, there are several species with unknown status that still require further investigation (Ho pers. data).

This work provides an opportunity to underscore the importance of not relying solely on previously published works but also examining type material in museum collections, even for well-known species.

### ﻿Material examined

***Lestrolepisjaponica***: **Holotype.** ZMUT 2013 (159.5), Sagami Bay, Japan. Paratype. ZMUT 2014 (ca. 180), same as holotype. **Non-types.** HUMZ 71212 (1, 183), Fukushima, Japan, trawl net, 200–300 m, 8 Nov. 1977. HUMZ 71248 (1, 243), Hayakawa Port, Odawara, Kanagawa, Japan, 7 Nov. 1977. HUMZ 71844 (1, 193), HUMZ 71845 (1, 296), HUMZ 71846 (1, 184), HUMZ 71847 (1, 213), HUMZ 71848 (1, 186), Hayakawa Port, Odawara, Kanagawa, Japan, 5 Nov. 1977. HUMZ 134997 (1), 38°35'30.0"N 141°59'48.0"E, off Minamisanriku, Miyagi, Japan, bottom trawl, 331 m, 17 Oct. 1994. HUMZ 163755 (1, 120), 38°24'32.4"N, 142°04'13.2"E Miyagi, 461 m, 12 Oct. 1999. HUMZ 163832 (1, 123), HUMZ 163833 (1, 125), Miyagi, 38°23'09.6"N, 142°07'27.0"E, 552 m, 26 Oct. 1999. HUMZ 206777 (1, 162), 38°24'57"N, 142°02'50.4"E, Miyagi, Japan, 425–430 m, 30 Oct. 2009. HUMZ 206833 (1, 140), 38°22'13.2"N, 142°03'13.8"E, Miyagi, bottom trawl, 412 m, 9 Oct. 2009. HUMZ 209243 (1, 92), 39°35'24.6"N, 142°31'E, 890-897 m, 7 Oct. 2010. HUMZ 214579 (1, 134), 39°05'15.6"N, 142°09'34.8"E, off Tohoku, 398–412 m, 19 Oct. 2011. HUMZ 222513 (1, 183), Miyagi, 38°52'01.2"N, 142°04'24.6"E, 357 m, bottom trawl, 23 Oct. 2013. HUMZ 226859 (1, 155), 39°01'59.4"N, 142°12'55.8"E, off Kesennuma, Miyagi, Japan, bottom trawl, 575 m, 23 Oct. 2015. HUMZ 226876 (1, 146), HUMZ 226877 (3), 37°36'56.4"N, 141°50'31.8"E, off Souma, Fukushima, Japan, 380 m, 5 Nov. 2015. HUMZ 226942 (2), 37°43'N, 141°53'51"E, off Souma, Fukushima, Japan, bottom trawl, 411 m, 29 Oct. 2015. NSMT-P13816 (2, 147–154), Suruga Bay, Shizuoka, Japan, 17 Nov. 1968. NSMT-P48916 (1, 168), south of Sagami Bay, Japan, 4 Nov. 1995. NSMT-P48931 (1, 154), off Tohoku, northern Japan, trawl, 5 Nov. 1995. NSMT-P58786 (3, 75–130), 39°00'36"N, 143°32'06"E, off Pacific coast between Miyagi Pref. and Iwate, 550–578 m, Japan, mesopelagic trawl, 29 Jul. 1996. NSMT-P 58787 (1, 100), 39°00'36"N, 143°32'06"E, between Miyagi Pref. and Iwate, 650–677 m, mesopelagic trawl, 29 Nov. 1996. NSMT-P58788 (1, 115), NSMT-P58789 (1, 93), 39°02'24"N, 143°30'07.2"E, between Miyagi Pref. and Iwate, northern Japan, 650–677 m, mesopelagic trawl, 30 Jul. 1996. NSMT-P65464 (1, 176), 37°45'54"N, 142°09'32"E, off Fukushima, northern Japan, 647–676 m, otter trawl, 19 Oct. 2002. NSMT-P65466 (1, 136), 36°53'45.6"N, 141°33'43.2"E, off Ibaraki, Japan, 495–530 m, otter trawl, 20 Oct. 2002. NSMT-P67563 (1, 158), 31°20'39.1"N, 128°10'53"E, southern Japan, 392 m, otter Trawl, 8 Nov. 2003. NSMT-P67589 (1, 237), 28°59'47"N, 127°09'21.2"E, Ryukyus, 350 m, otter Trawl, 5 Nov. 2003. NSMT-P91547 (1, 170), 38°21'32.4"N, 141°56'24.0"E, off Miyagi, Japan, 280 m, trawl, 3 Oct. 2007. NSMT-P102802 (1, 169), 36°58'26.4"N, 141°25'43.3"E, off Fukushima, Japan, 251-252 m, otter trawl, 26 Oct. 2006. Also listed as *Lestrolepisnigroventralis* in Ho et al. (2019a), including the type series.

***Lestrolepisphilippina***: **Holotype.** USNM 92323 (118.2), Varadero Bay, southern Luzon, Mindoro, Philippine, 22 Jul. 1908. Paratypes. USNM 93414 (2, 109.6–125), Noble Point, Tulayan Island, Sulu, Philippine, 283 m, 15 Sep. 1909. CAS-SU 14970 (1), USNM 135257 (2, 121–128), Anchorage, Dupon Bay, Leyte, Philippine, 17 Mar. 1909. **Non-types.** HUMZ 146645 (1, 228), 25°37'16.8"N, 126°05'21"E, Miyoko, Okinawa, 388–394 m, 2 Aug. 1994. HUMZ 222365 (1, 153), HUMZ 222366 (1, 165), HUMZ 222367 (1, 180), HUMZ 222368 (1, 185), HUMZ 222462 (1, 144), Dong-gang, Taiwan, 7 Nov. 2013. NSMT-P59709 (2, 87–96), 35°50'38.4"N, 152°59'49.2"E, 0–20 m, trawl, 24 May 1995. NSMT-P67590 (2, 185-232), 28°59'47"N, 127°09'21.2"E, East China Sea, 350 m, otter Trawl, 5 Nov. 2003. NSMT-P94628 (8, 123–160), Sagami Bay, southern Japan, set net, Tokyo Sea Life Park, 15 Nov. 1988. NSMT-P115090 (1, 138.2), NSMT-P115091 (1, 154.5), Dong-gang fishing port, Taiwan, 17 Oct. 2013, G. Shinohara et al. NSMT-P115637 (1, 142), Dong-gang fishing port, Taiwan, 19 Oct. 2013. NSMT-P128174 (1, 268.8), off Amami-Oshima Is., Ryukyus, Japan, Yamakawa, 26 Jun. 1972. NSMT-P128175 (1, 226.1), off Amami-Oshima Is., Ryukyus, Japan, 26 Jun. 1972. Also listed as *L.japonica* in Ho et al. (2019a)

## Supplementary Material

XML Treatment for Lestrolepis japonica

XML Treatment for Lestrolepis philippina

## ﻿Additional information

### Author ORCIDs

Hsuan-Ching Ho https://orcid.org/0000-0003-1154-601X

### Data availability

All of the data that support the findings of this study are available in the main text.
